# A Fast SVM-Based Tongue's Colour Classification Aided by *k*-Means Clustering Identifiers and Colour Attributes as Computer-Assisted Tool for Tongue Diagnosis

**DOI:** 10.1155/2017/7460168

**Published:** 2017-04-20

**Authors:** Nur Diyana Kamarudin, Chia Yee Ooi, Tadaaki Kawanabe, Hiroshi Odaguchi, Fuminori Kobayashi

**Affiliations:** ^1^Embedded System Research Laboratory, Department of Electronics System Engineering, Malaysia-Japan International Institute of Technology, Kuala Lumpur, Malaysia; ^2^Oriental Medicine Research Center, Kitasato University, Minato, Japan

## Abstract

In tongue diagnosis, colour information of tongue body has kept valuable information regarding the state of disease and its correlation with the internal organs. Qualitatively, practitioners may have difficulty in their judgement due to the instable lighting condition and naked eye's ability to capture the exact colour distribution on the tongue especially the tongue with multicolour substance. To overcome this ambiguity, this paper presents a two-stage tongue's multicolour classification based on a support vector machine (SVM) whose support vectors are reduced by our proposed *k*-means clustering identifiers and red colour range for precise tongue colour diagnosis. In the first stage, *k*-means clustering is used to cluster a tongue image into four clusters of image background (black), deep red region, red/light red region, and transitional region. In the second-stage classification, red/light red tongue images are further classified into red tongue or light red tongue based on the red colour range derived in our work. Overall, true rate classification accuracy of the proposed two-stage classification to diagnose red, light red, and deep red tongue colours is 94%. The number of support vectors in SVM is improved by 41.2%, and the execution time for one image is recorded as 48 seconds.

## 1. Introduction

**Figure 1:**
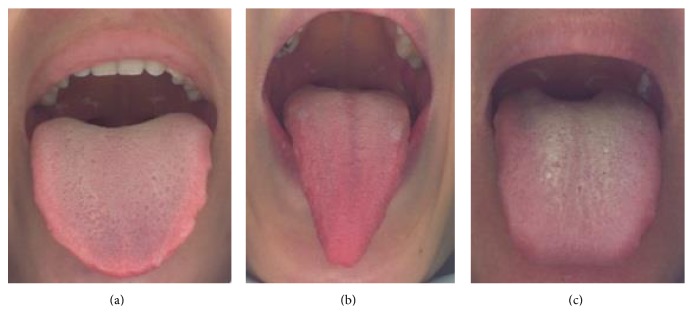
Tongue colour samples: (a) light red, (b) red, and (c) deep red.

Tongue diagnosis is said to be the most active research in the advancement of complementary medicine compared to other diagnosis fields such as pulse and abdominal palpation [1]. For several decades, the objectification of tongue's colour, texture, and geometry analysis as well as pathological feature disease correlations have been researched thoroughly to achieve standardization in clinical practice and to improve the existing technology of computerized tongue image analysis device. In traditional east-Asian medicine, precise tongue colour quantification is essential to predict patients' illness caused by physical and mental disharmony. Hence, tongue colour provides beneficial information on blood congestion, water imbalance, and psychological problems [2]. In *Kampo medicine* as well as traditional Chinese medicine (TCM), tongue colour is mainly classified into three colors such as red, light red, and deep red as illustrated in Figure 1.

In 2010 and 2013, a tongue colour gamut descriptor has been proposed by several researchers using one class SVM [3, 4]. This proposed work suggested that the tongue colour gamut is very narrow and comprises of different types of identical colour; thus, there are many overlapping and similar pixel values that exist. By using the naked eye, the colour information on different regions of a tongue body (or substance) might look almost similar. Nevertheless, when we clustered the tongue body image into distinguished pixels using clustering algorithm, there exist several clustered regions with different colour information on a tongue. These clustered colour information is very useful in order to determine what are the most contributing colour or area on a tongue body for tongue colour evaluation and diagnosis. However, in order to choose the most informative clustered pixels to be used as input features in a classifier, an effective feature selection method is necessary. According to [5], there are several problems during preprocessing and data selection that can reduce the performance of a classifier:

- Impossible features or values have been inputted as training examples.
- Several values have not been inputted during training (missing values).
- Redundant or irrelevant data have been included as training examples.

Similar researches in [6–9] have been discussed to sift out redundant information from the samples used in the classifier during data selection. Nevertheless, some of these works did not consider the basis theory of the sample selection and the possibility of misclassification or misclustering during sample selection. Moreover, too many features included in the training examples have led to complex descriptive feature mapping in the classifier [10]. Currently, there are many researches in computerized tongue image analysis system that utilized machine learning techniques aiming to have better accuracy rate and lesser runtime. Yet, most of the journals or research works have been reported on a trade-off between these two parameters (computation time and accuracy). Several works only reported on the optimization of classification accuracy but not the execution time [10–13].

Therefore, creating a classifier by taking into account the most informative or meaningful features to improve the response time and generalization ability is crucial. This paper presents a two-stage classification system for tongue colour diagnosis aided with the devised clustering identifiers and proposed colour range that can improve the classification accuracy and classifier's response time. Data selection method in our case (*k*-means clustering) is based on tongue colour pattern and area using traditional medicine perspective. The purpose of these clustering identifiers is to collect only meaningful tongue features to reduce the classifier runtime via the reduction of support vectors and outliers. Besides, red colour range in the second stage of classification reduces the possibility of overlapping pixels in the new examples that can boost up the classification accuracy. To the best of our knowledge, this is the latest tongue colour diagnosis system that promotes high accuracy and fast response time classification system which considered the most contributing colour and area analysis using *k*-means, SVM, and colour range. This novel two-stage classification system outperformed the conventional SVM by 20% in terms of computational time and 15% in terms of classification accuracy.

## 2. Previous Works

Feature extraction or data transformation is a process of transforming a raw feature data into quantitative data structure or patterns for training accessibility [14]. Feature detection and extraction is an essential procedure in tongue diagnostic system after segmentation procedure as some of the tongue features accumulated beneficial information related to the internal body system and health. During tongue manifestation, the medical practitioners examined the tongue's features such as tongue coating, teeth marks, prickles, purple spot and blue spot, or any abnormal features on the tongue to predict the condition of their patient. For instances, teeth marks on a tongue can be related to the dehydration of fluid in the body, substance with existence of purple spot can be related to the blood congestion inside the body, too many obvious prickles are related to the appendicitis [15], and more feature disease or disharmony state of the body correlations can be predicted. Hence, an accurate feature detection or extraction algorithm is crucial to examine the image in the feature's region comprehensively to acquire useful information in terms of its colour or texture.

To establish the standard of tongue diagnosis, the standardization fundamental in defining the most suitable informative features for any significant diseases is also essential. There are several reported works using hyperspectral images to attain meaningful tongue features for further analysis such as coating colour and sublingual veins [16–18]. Even though the hyperspectral image sensing is insensitive to various illumination and albedo effects, the high cost of the hyperspectral camera is a limitation. Since decades, the objectification of tongue colour, texture, and geometry analysis as well as pathological feature disease correlations have been researched comprehensively to establish a computerized tongue diagnosis device. Moreover, to determine the most important features that can exclusively contribute to improve performance of the classifier is very crucial. Some related works are reported in [6–9] using hybrid classifiers to sift out several redundant features and adopted only the most meaningful instances to boost up the performance of the classifier in terms of its response time and classification accuracy. Through this motivation, this technique will reduce the number of training examples and lead to the number of support vectors reduction, thus speeding up the classifier's response time. However, some of these works have not considered the possibility of misclassification or misclustering during sample selection. In addition, the number of clusters produced by *k*-means clustering is always the same as the number of classes produced by SVM; hence, it cannot be applied in certain cases where SVM classes are less than the clusters of *k*-means which may occur in some classification problems. To the best of our knowledge, none of them have been implemented on image applications.

Because there is abundance of tongue features or pathological details that have been accumulated via thousands of images progressively, the innovation in decision support system and intelligent image analysis has evolved for accurate and fast classification and diagnosis. In general, there are three learning algorithms which have been implemented using machine learning such as supervised, unsupervised, and semisupervised algorithms. By utilizing these algorithms, an accurate classification system to classify the most informative features with high generalization ability is desired. There are several works reported in feature disease classifications aiming to predict the mapping relationships between tongue features and diseases [10, 11, 15, 19–21]. There are several researches that utilized neural network in categorizing the tongue features [18, 22–24]. Nonetheless, the two most applied classifiers in tongue diagnosis field are Bayesian network classifier [10, 25] and SVM-based classifier [3, 11–13, 26, 27]. Even though there are sufficient training examples from the textural and chromatic properties of a tongue used in the classifier, the accuracy in some reported works needs to be improved [10, 28].

In [26], an SVM-based algorithm called transductive support vector machine (TSVM) is proposed by combining the labelled and unlabelled samples of tongue features as training examples to reduce the human labour and improve the classifier's accuracy since the unlabelled samples help to provide much more classification information during the training process. Nevertheless, there are several classification problems of ambiguous separating boundaries between the classes. This is because there is no model selection method has been made prior to the classification during the training process. The more unlabelled samples are included, the noisy the data will be. Hence, the study on the selection method of unlabelled samples remained as a future research. To compare the performances among classifiers, this research paper has investigated five types of machine learning algorithms to foresee the performance of these algorithms in terms of tongue's features and classification [11]. Five different machine learning algorithms including ID3 (based on decision tree), J48 (based on decision tree), naive Bayes (based on Bayesian network), BayesNet (based on Bayesian network), and sequential minimal optimization (based on SVM) were applied to a tongue dataset of 457 instances. Their comparison results have shown that the SVM-based algorithm has relatively the best performance. However, with the abundance of accumulated tongue features in the near future, the limitations of conventional SVM algorithm are on its speed and size. For a similar generalization performance, SVM response time is slower than other neural network algorithms [29]. The computational complexity that is linear with the number of support vectors is an unsolved problem [30]. To date, the issue on how to choose a good kernel functions in a data-dependent way [31] and how to control the selection of support vectors has been researched thoroughly especially in a noisy and continuous data [32].

## 3. Materials and Method

### 3.1. Clinical Data Collection

There are a total of 300 tongue images after coating eliminations have been accumulated during clinical practice in the Oriental Medicine Research Centre, Kitasato University in Japan. All tongue images in this proposed research were taken by tongue image analyzing system (TIAS) that was invented by the Chiba University, Japan. TIAS is a closed box acquisition system that is used to capture the tongue image under stable condition in terms of illumination condition and tongue's position. There are several components implemented in TIAS such as

- halogen lamps as illuminators with high colour temperature to acquire adequate tongue colour information,
- integrating sphere which is a hollow cavity shaped with coated interior to produce equal distribution of light rays on a tongue,
- 1280 × 1024 pixels high-speed charged couple digital (CCD) camera to capture high-resolution 24 bit RGB (redness, greenness, and blueness) tongue images,
- 24-colour chart for colour correction purposes.

All the images implemented underwent colour correction procedure to maintain high colour reproducibility outcomes. Around 300 tongue images after coating elimination in [33] are used in *k*-means clustering procedures and 600 features or instances of clustered results or what we called clustering identifiers are used as training examples in SVM.

### 3.2. Fast SVM Aided by *k*-Means Clustering Identifiers and Colour Attributes

**Figure 2:**
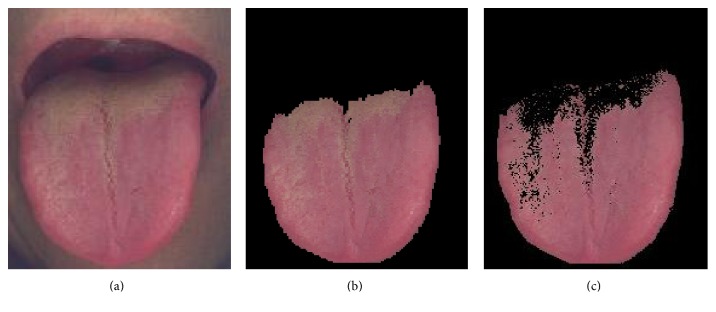
(a) Raw image, (b) image after segmentation, and (c) image after coating removal.

This section describes our full procedures on our proposal of two-stage classification system by implementing an SVM classifier aided with clustering identifiers and red colour range. The preclassification starts with the most contributing colour and area analysis using *k*-means clustering to develop clustering identifiers that can be used as training examples to recognize tongue colour in SVM. This automatic classification system is proposed to classify tongue images into light red tongue, red tongue, and deep red tongue. In the first-stage classification, tongue images after segmentation and coating removal such as in Figure 2 are fed to *k*-means clustering algorithm with *k* = 4 to determine the most contributing colour and area on the tongue image.

The outcome of the clustering procedure is the cluster image that is divided into background, red/light red, deep red, and region with transitional pixel clusters. The two most informative clusters or clustering identifiers are red/light red and deep red clusters. These two clustering identifiers will be used as training examples in SVM to preclassify the deep red tongue or red/light tongue. In the second-stage classification or final classification, the red/light red tongue images are further classified into red tongue and light red tongue based on the red colour range derived from our databases. The detail on how we choose the most informative clusters will be discussed in the next section.

#### 3.2.1. The Most Contributing Colour and Area Analysis Using *k*-Means Clustering Algorithm

The most important step in our proposed classification technique is a sampling strategy of bag-of-features reduction (or also known as feature selection) by applying clustering algorithm to define the most contributing colour and area on tongue images. Feature selection is a procedure of selecting an informative subset of nonredundant features among the original or transformed ones usually for efficiency purposes [34]. This technique is mainly proposed to define the most meaningful colour regions or area (or clusters) that can contribute to diagnose the tongue colour (red, light red, and deep red). Moreover, it is also can be considered as a down sampling technique to eliminate the less contributing colour so that the computational complexity is reduced. Clustering is the process of partitioning a group of data points into a small number of groups. The goal is to assign a data label to each data point and separate the data points to every cluster that depends on the similar label. Given a set of *n* data points in *d*-dimensional space *R*^*d*^ and an integer *k*, *k*-means clustering determines a set of *k* centre points in *R*^*d*^ such that the mean squared distance from each data point to its nearest centre point is minimized. In image processing analysis, each pixel in the input image was treated as an object that has a location in space. *k*-means clustering is usually implemented to solve the image segmentation or feature extraction and feature classification. In our proposed work, *k*-means clustering algorithm has been implemented with *Lab* colour space to produce informative cluster information and to identify some hidden patterns that can be used as training pixels in SVM to diagnose the tongue colour.

In east-Asian medicine perspective, the tongue accumulates several valuable information regarding the properties, location, and development and prognosis of a disease [35]. The tongue is usually diagnose by observing its bilateral edge regions [2]. Nevertheless, according to TCM, there are five regions of the tongue that can be useful to relate directly or indirectly to the internal organs such as bilateral edges (related to liver and gallbladder), tip (heart and lung), centre (spleen and stomach), and root (kidney). To this motivation, we want to determine the most contributing colour and area on a tongue body that can be used to diagnose the tongue colour accurately. Apart from tongue's root regions, we have discovered that by using clustering algorithms, the tongue regions are distinguished via the edges, the tip of the tongue, and the centre of the tongue. Moreover, we have observed that most of the pixels are clustered at the tip, bilateral edge, and the whole tongue's region (tip, edges, and centre). Hence, we have adopted the two most informative clusters or also known as clustering identifiers which represent the pixel distribution around the tongue's tip and edges which we called the highest colour distance from black pixel (0, 0, 0) and the highest number of pixel cluster which has been distributed sparsely across the tip, edges, and centre of a tongue. We named them as maximum colour distance identifier and maximum pixels' coverage area identifiers to identify the red/light red cluster and deep red cluster, respectively. This procedure was done along with the verification of several Oriental medicine practitioners at Kitasato University, Japan.

The *k*-means clustering formula is described using cluster centroid detection given by

(1) $$\begin{array}{c} \Delta_{distance\;metric}=\sqrt{{\left({L_{1}}^{*}-{C_{1}}^{*}\right)}^{2}+{\left({a_{1}}^{*}-{C_{2}}^{*}\right)}^{2}+{\left({b_{1}}^{*}-{C_{3}}^{*}\right)}^{2},} \end{array}$$

where (*C*_1_^∗^, *C*_2_^∗^, *C*_3_^∗^) is the centroid of each cluster.

We have tested several possible number of clusters and observed the outcomes with the practitioner's recommendation. By using *k* = 3, the mixture of red, light red, and deep red pixels on one cluster was detected by observing the centroid value of each cluster and the pixels are vaguely distinguished. Whilst, by using *k* = 5, the redundant clusters sharing similar average colour were detected or, in other words, the colour distance between each cluster is not significant. After several experiments, we have chosen *k* = 4 because tongue substance usually comprises of three main colours which is red, light red, and deep red. The other one cluster left is reserved for a background. Moreover, by looking at the perspectives of pixel's distribution around four tongue regions (left and right edges, tip, and the centre) mentioned previously, we found that *k* = 4 is the most accurate cluster number that characterised the distribution. The practitioners confirmed all clustered images on an IPS (in-plane switching) monitor (ColourEdge CG246, EIZO®). The development of clustering identifiers can help in boosting up the performance of SVM via the reduction of noisy pixels and outliers in the training data.

#### 3.2.2. *k*-Means Clustering Identifiers

Based on our observation during the process of determining the number of cluster, *k* using resulting clusters of *k*-means, we have recognized two hidden patterns or we called it as identifiers to identify the clusters that contained deep red region and red/light red region. These identifiers are devised based on (i) maximum colour distance from black pixel (0, 0, 0) and (ii) maximum pixel's coverage area. Moreover, based on the prelabelled images beforehand, the output clusters from the *k*-means were also measured in terms of their average colour value in *Lab* colour space to develop the red colour range identifier to be used in the second-stage diagnosis.

Maximum colour distances from black pixel or chromatic intensity are determined based on (2) as

(2) $$\begin{array}{c} {∆}_{colour\;distance}=\sqrt{{\left({L_{2}}^{*}-{L_{1}}^{*}\right)}^{2}+{\left({a_{2}}^{*}-{a_{1}}^{*}\right)}^{2}+{\left({b_{2}}^{*}-{b_{1}}^{*}\right)}^{2}}, \end{array}$$

where (*L*_1_^∗^, *a*_1_^∗^, *b*_1_^∗^) is (0, 0, 0). Using this identifier, pixels with more red and less blue (higher *a*^∗^and *b*^∗^) concentration (red and light red) can be easily detected compared to pixels with less red and more blue (lower *a*^∗^and *b*^∗^) concentration because these pixels have longer distance from the black pixel. In our *Lab* colour analysis on substance colour after coating removal, red and light red tongue has been observed to have a domination of *a*^∗^, *b*^∗^ (chromatic) pixels compared to deep red tongue that was influenced by black colour as their distance to black pixel is nearer [33]. Hence, after series of training procedures in SVM, this identifier has the best accuracy to classify red/light red tongue.

**Figure 3:**
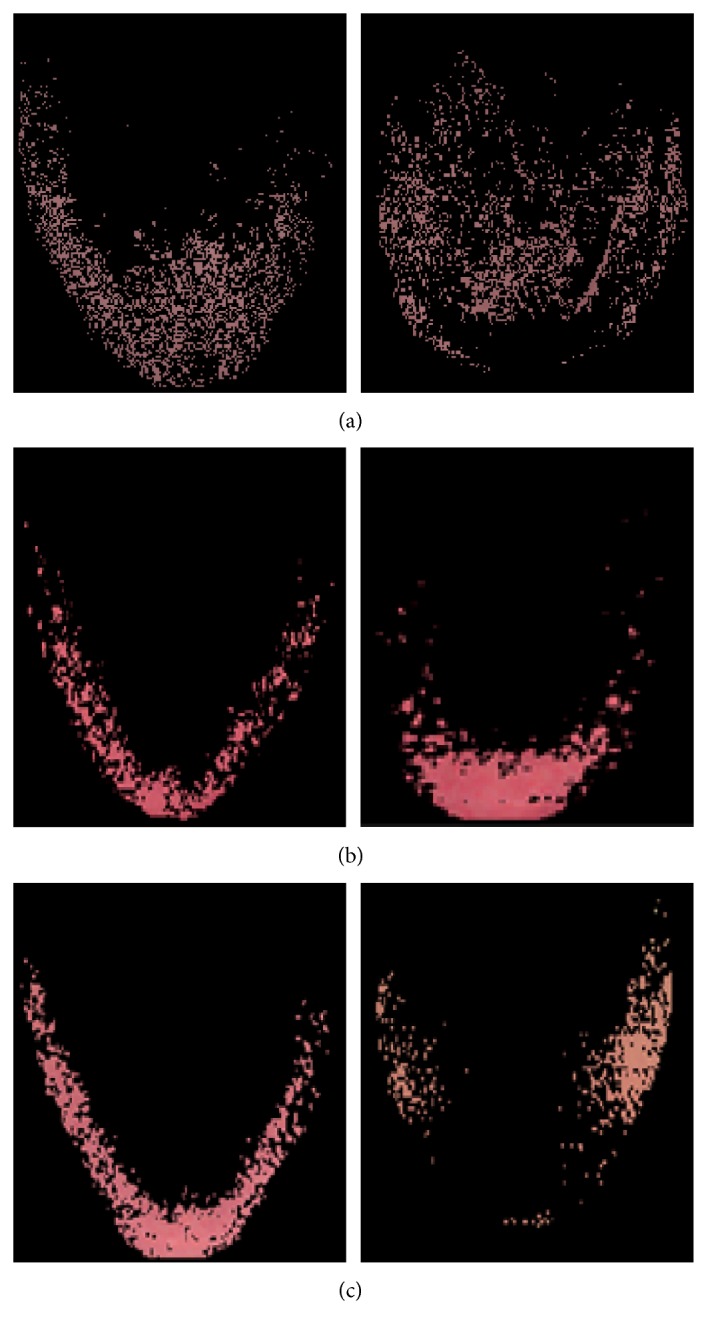
(a) Deep red cluster identified by maximum pixels' coverage area identifier and (b) and (c) red/light red clusters identified by maximum colour distance identifier.

Moreover, maximum pixels' coverage area formula can be deduced as in

(3) $$\begin{array}{c} A_{np}=w^{\prime }\times h^{\prime }, \end{array}$$

where all nonzero pixels are covered by the area where *w*′ ≤ *w* and *h*′ ≤ *h*. In this equation, *A*_np_ is the number of nonzero pixels area in the tongue image after clustering, *w* is width area of tongue image before clustering, and *h* is the height area of tongue image before clustering. Samples of the deep red clusters and red/light red clusters identified by our proposed clustering identifiers are as shown in Figure 3.

During the accumulation of average colour of every clusters in *k*-means procedure, the average colour of the maximum pixels' coverage area clusters is observed to have the least concentration of red (denoted by low *a*^∗^ and *b*^∗^ attributes) in *Lab* colour compared to other clusters. Hence, after series of training procedures in SVM, this identifier has the best accuracy to classify deep red tongue.

#### 3.2.3. Reduction of Support Vectors in SVM via Tongue Clustering Identifiers

This section describes the theory and fundamentals of our proposed SVM model aided with clustering identifiers to reduce the number of support vectors during training and testing procedures that will lead to a fast classification system with high accuracy. A support vector machine (SVM) is a supervised machine learning method that is defined by a separating hyper plane which can be used for classification of images. Given a set of labelled training data, the algorithm outputs an optimal hyper plane which predicts the new example to fall on which side of the gap. In the image processing concept, this training algorithm of SVM builds a model of mapping pixels and assigns them into one category or the other divided by a discriminative hyper plane. A good separation is achieved by the hyper plane that has the largest margin which describes the distance to the nearest training data point (support vectors) of any class. The larger the margin, the lower the generalization error of the classifier will be. The reason why SVM insists on finding the maximum margin hyper planes is that this optimization offers the best generalization ability. In other words, it compromises better classification performance (e.g., accuracy) on the training data of the future data. In addition, SVM can also perform a nonlinear classification using the kernel method by mapping their inputs into high-dimensional feature spaces implicitly. Besides, SVM is said to have high generalization ability for classification problem compared to other machine learning algorithms even though the input space is very high [36].

Currently, there are two types of approaches for multiclass SVM. The first method is by considering all the data using one optimization formula and the other one is by combining several binary classifiers together and finally opt for the one with the best optimization. According to this paper [37], the formulation to solve multiclass SVM problems in one step requires the number of variables to be proportional to the number of classes. Therefore, there must be several binary classifiers to be constructed or a larger optimization problem is needed. Hence, in general, it is computationally more expensive to solve a multiclass problem than a binary problem with similar number of training databases [38]. Nevertheless, when the training data becomes bigger, more time is needed for optimization. In [39], one of the limitations of SVM classifier is its computational inefficiency when there are millions of instances to be classified. However, this problem can be solved by breaking the core sets into a series of subsets. Through this motivation, we have proposed a two-stage classifier that can break a large optimization problem into a series of small problems (clusters) where each problem only involves a small number of informative pixels (small number of support vectors too) so that the optimization problems can be solved successfully. Solving these small variables will definitely save its response time whilst the generalization ability can be improved significantly.

Theoretically, the general binary (two classes) classification using SVM can be visualized in Figure 5. The motivation of our proposed algorithm is to have a margin that is as wide as possible to separate the training points between the two classes (deep red and light/red tongue) depending on the location of support vectors. The general equation of these hyper planes that separate these two categories can be described as

(4) $$\begin{array}{c} \begin{array}{cc} y_{i}\left(w^{T}x_{i}-b\right)\geq 1 & 1\leq i\leq n\\ \min\frac{1}{2}\;{\left\|w\right\|}^{2}\;s.t.\;y_{i}\left(w^{T}x_{i}-b\right)\geq 1, & i=1,2,\ldots ,n, \end{array} \end{array}$$

where *w* is the normal vector of the hyperplane, *b* is the bias, and *x*_*i*_ is the support vectors that lie on a feature space. In our case, the training input to SVM can be devised in pseudocodes given by Algorithm 1.

**Figure 4:**
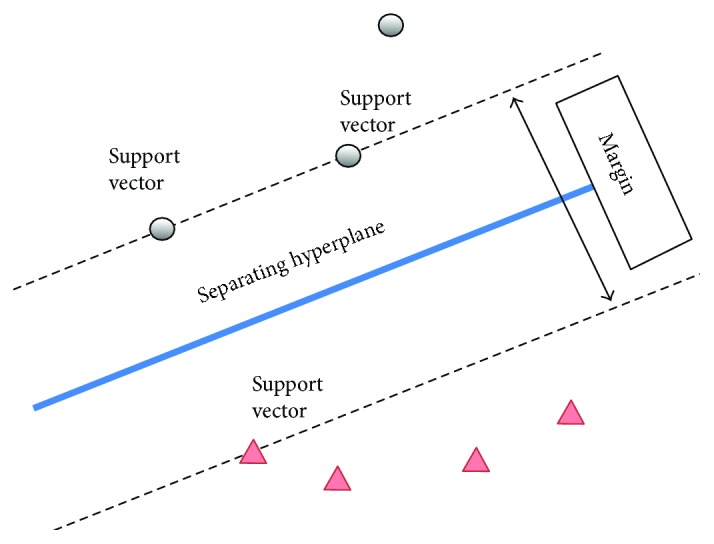
SVM concept of classification by constructed hyper plane.

Methodically, if we apply this (4) to the set of test data, there will be many hyper planes generated to classify the data with respect to two different constraints. The best choice of hyper planes is the one that has the largest separation between the two classes as illustrated in Figure 4. These *w* and *b* parameters are solved to determine the classifier. The maximum margin can be determined by those *x*_*i*_ which lie nearest to it. These *x*_*i*_ are called boundary points or support vectors. The complexity of the SVM algorithm is really depending on the number of support vectors. In other words, the cost function does not explicitly depend on the dimensionality of feature space and the number of training samples. In soft margin cases, we need more training samples to get better generalization ability and lesser number of support vectors. In SVM, there are many types of kernel such as linear, Gaussian, quadratic, and polynomial. The kernel is defined as the inner product in the feature space. Kernel trick in SVM learns linear decision boundary in a high dimension space without explicitly working on the mapped data. The linear kernel is the simplest example of a kernel and is obtained by considering the identity mapping for the feature space to satisfy *φ*(*x*) = *x* or in formulation *k*(*x*, *x*′) = (*x*^*T*^, *x*′).

**Figure 5:**
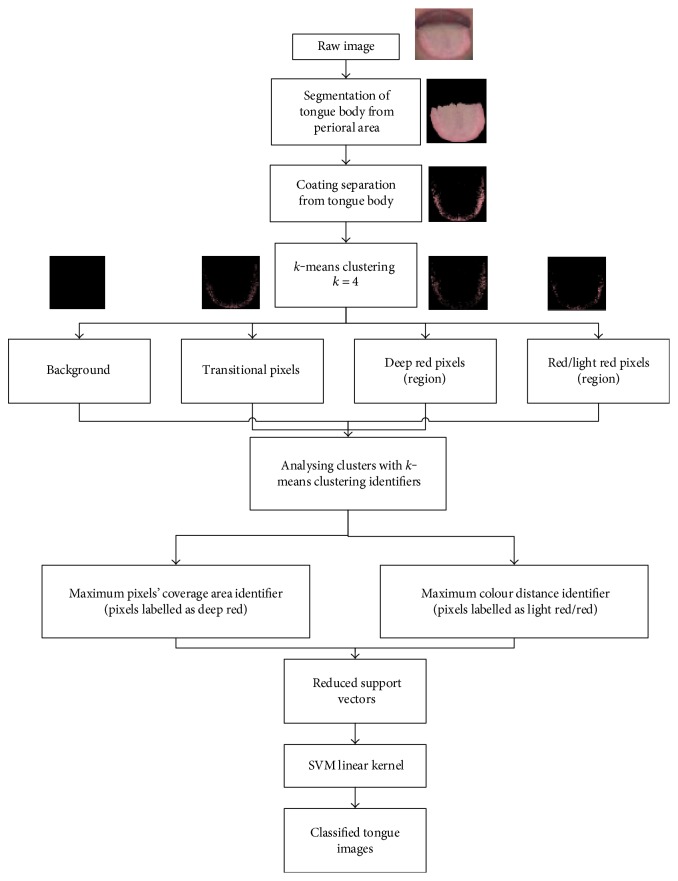
Flowchart of our proposed first-stage classification.

The down sampling procedures or sample selection method for training purposes before classification is essential to reduce classifier's burden and complexity. Since the tongue colour is very narrow [40], there are many overlapping pixels of similar colour exist. In other words, the distributions of tongue image pixels are usually nonlinear and continuous. In our proposed work, we have discovered that the most contributing colour and area analysis on the tongue by *k*-means clustering can be used to extract meaningful and informative colour region on the tongue body so that the redundant pixels can be eliminated. However, not all clusters produced by our proposed *k*-means clustering algorithm are relevant to be fed as training examples in SVM. To this motivation, we have developed two tongue clustering identifiers to be fed as training examples in SVM and they are called as maximum pixels' coverage area identifier and maximum colour distance identifier. An outline of our first-stage classification system aided by these clustering identifiers as training examples to distinguish the deep red and red/light red group of tongue can be visualized in Figure 5.

After series of training procedures, we have discovered that maximum colour distance identifier has the best ability to classify deep red tongue and maximum pixels' coverage area identifier has the best ability to classify red/light red tongue based on the loss function formula calculated using labelled tongue colour image databases. By using these proposed clustering identifiers, the numbers of overlapping pixels and misclassified points or outliers between the boundaries have been greatly reduced; hence, the number of support vectors is also reduced. This reduction of overlapping pixels promotes distance maximization (margin maximization) of separating hyper plane for better generalization ability. The implementation of our proposed tongue identifier as training examples is significant as it also lead to minimization of outliers that can prevent over fitting during the classification process. Moreover, SVM treats all training points equally; hence, both the noisy points and outliers will have negative impacts on the accurate classification [41]. Methodically, in order to classify some training points using an SVM model, the dot product of each support vector has to be computed with every test point. In other words, the SVM model did faster classification with fewer number of support vectors and vice versa. Thus, the computational complexity of our proposed classifier model aided with the clustering identifier model has been reduced via the reduction in the number of support vectors. The detailed measurement analyses and results of our proposed classification method will be discussed in the Experimental Results and Discussion. For SVM accuracy measurement, we have estimated the classification rate terminology via the loss function formula given by

(5) $$\begin{array}{c} ACC≅\left(\frac{n_{red/light\;red}+n_{deep\;red}}{N}\;\right)\times 100, \end{array}$$

where ACC is the estimated successful classification accuracy rate, *n*_red/light red_ is the number of red/light red tongue images that have been correctly classified, *n*_deep red_ is the number of deep red tongue images that have been correctly classified, and *N* is the total number of tongue images used in classification.

### 3.3. Red Colour Range in *Lab* Colour Space

This section describes the second stage of classification where red colour range is used as a final classifier between red/light red groups of tongue after first classification is done. If the new example of tongue is classified as deep red tongue after the first stage of classification, then it will not be classified further using this red colour range. Nevertheless, if the new example is classified as red/light red tongue in the first stage of classification, then, it has to be classified further using red colour range for final verification. The measurement of red colour range is done during the clustering procedures where we have accumulated hundreds of average red and light red tongue colour clusters which are labelled clinically beforehand by the practitioners' naked eye as red and light red tongues. By naked eye, red and light red tongues look very similar because the colour range of chromatic value (*a*^∗^ and *b*^∗^ attributes) is relatively similar for red and light red tongues [33], but technically, the value of luminance attributes (*L*^∗^) is distinguishable. The red colour range in *Lab* colour space is summarized in Table 1.

After the first-stage classification, red/light red cluster will be tested using red colour range defined in Table 1. The new example of red/light red cluster should satisfy the range value of every attribute (*L*^∗^, *a*^∗^, *b*^∗^) in the table above. Red/light red cluster which does not fit the range of *a*^∗^, *b*^∗^ value in Table 1 will be further classified based on luminance, *L*^∗^ value afterwards. Subsequently, the user will get the final result of the tongue colour whether it is red or light red tongue. The red colour range pseudocodes were given by Algorithm 2.

**Figure 6:**
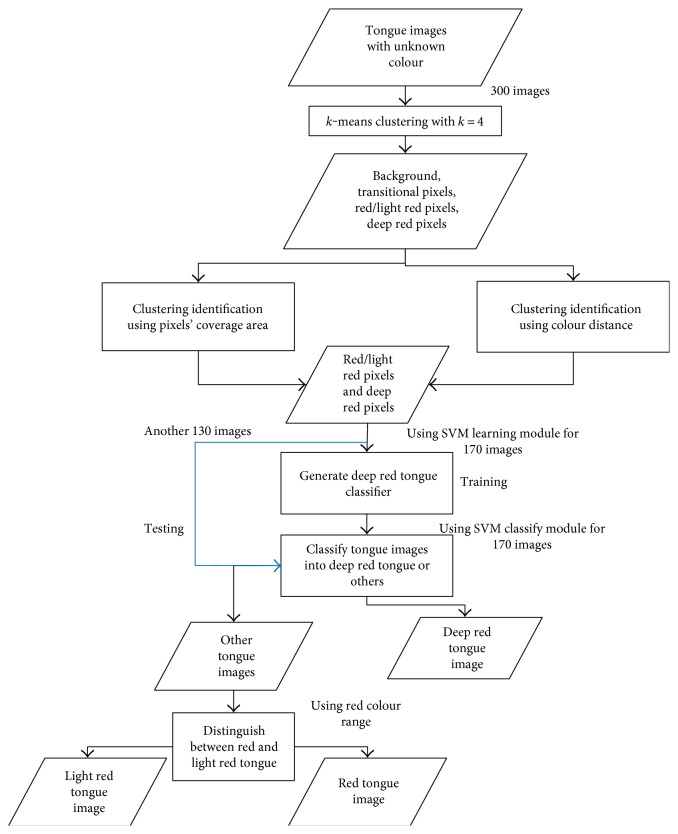
The outline of proposed computerized tongue colour diagnosis system.

The detailed procedures of our proposed tongue colour diagnosis system aided with the proposed identifiers and red colour range are illustrated in Figure 6.

## 4. Experimental Results and Discussion

### 4.1. Performance Analysis

This section discusses the experimental results and the performances of our proposed two-stage classification system using SVM model with clustering identifiers and red colour range. We have involved over 300 images and 600 features of our clustering identifiers as training examples in SVM to diagnose the red, light red, and deep red tongue colours automatically. These tongue images were taken by tongue image analyzing system (TIAS) on hundreds of outpatients in the Oriental Medicine Research Centre, Kitasato University in Japan, and each of the tongue colour was validated and labelled beforehand by several medical practitioners. The SVM algorithm is run in MATLAB environment with Intel® Core™ i7-3820CPU @3.60GHz. As a comparison, we have implemented raw images after segmentation and coating removal as training examples without clustering identifiers. Several types of kernels such as linear, Gaussian radial basis function (RBF), quadratic, and polynomial kernels have been tested. However, without clustering identifier, the accuracy rate measured was not convincing. The rows labelled as “Conventional SVM” in Table 2 summarizes the performance of the SVM algorithm tested with different kernels in terms of their classification accuracy and execution time. The last row in Table 2 shows the performance of neural network algorithm in classifying tongue colours whereby only 70.6% best classification rate was achieved.

Methodically, we have divided the experiment into two stages, SVM classifier and classification based on *Lab* colour ranges. The SVM is used to discriminate between the deep red and red/light red tongue colours aided by the proposed clustering identifiers. The best estimated classification rate attained is 89% when the linear kernel is being used as summarized in the rows labelled as “SVM with clustering identifiers” in Table 2.

Based on the measurement result by SVM on 300 images, we have deduced that linear kernel is the best kernel that can successfully separate the light red/red and deep red tongues using combination of maximum colour distance and pixels' coverage area identifiers. By implementing the result of *k*-means clustering algorithm that are based on colour distance and pixel's coverage area, the information of training and testing images to be tested by SVM had been simplified. Redundant pixels inside a tongue image after segmentation and coating removal that are not useful have been filtered out via our proposed clustering identifiers. By using these identifiers, there is a significant difference in classification boundary between these two classes (red/light red and deep red). In other words, the solution of SVM has been simplified.

However, a low classification accuracy have been observed in light red and red tongue classification by using maximum colour distance identifier in SVM because these two colours look very similar to the naked eye. Nevertheless, during the colour analysis measurement, the difference in light red and red tongues was observed via each of the *Lab* colour attributes: *L*^∗^, *a*^∗^, and *b*^∗^ statistically. Therefore, in order to devise a reliable and precise diagnosis system, we have derived the red colour range from *Lab* colour space for red and light red tongues to be used to distinguish these two colours in second-stage classification such that overall classification accuracy is high for the whole tongue colour diagnosis system. These colour attributes were accumulated during the clustering procedures.  Table 3 showed the comparison of accuracy between chromatic and luminance attributes between red and light red tongues. This chromatic and luminance attributes pattern indicator (*Lab* attributes) does not apply to deep red tongue because deep red tongue can be recognized by the first-stage classifier.

As can be seen in Table 3, low accuracy was recorded if only chromatic or colour attribute range (range for *a*^∗^ and *b*^∗^ only) was used to recognize the light red and red tongue colours. In the light red and red tongues, several pixel values of *a*^∗^ and *b*^∗^ are very similar; hence, there are some overlapping pixels between these two colours. Therefore, luminance can distinguish the light red and red more accurately. Here, chromatic with luminance attributes from the *Lab* colour space gave the highest accuracy in red and light red tongue second-stage classification which is 95%. In total, the estimated classification accuracy using our proposed two-stage classifier was recorded as 94%. Therefore, we have developed the intelligent diagnosis system that can predict and diagnose the tongue based on its colour and also can be an assisted tool for the practitioner during the clinical practices.

## 5. Conclusion

In this work, we have proposed a two-stage classification method to diagnose three tongue colours: red, light red, and deep red. The proposed automatic colour diagnosis system is very useful for early detection of imbalances condition inside the body. According to traditional medicine perspectives, light red tongue is considered normal; red tongue is always associated to excess of heat, dehydration, hemoconcentration, or irritability; and deep red tongue is associated with blood stagnation, coldness, and so forth. The first-stage classifier is mainly based on SVM aided by *k*-means clustering identifiers: maximum colour distance identifier and maximum pixel's coverage area identifier. These two identifiers have been employed to discriminate between the red/light red with deep red tongue colour with measured classification accuracy of around 89%. To further obtain the separation between the light red tongue and red tongue accurately, red colour range using *Lab* colour space were introduced for red and light red clusters. These colour attributes were measured using average cluster value of red and light red clusters during clustering process. The accuracy of second-stage classification using red colour range has been recorded as 95%. Finally, using our proposed two-stage classifier, the overall estimated successful classification rate to discriminate red, light red, and deep red tongue colours is 94%. The whole algorithm execution time is around 48 seconds to diagnose one tongue image which offers fast processing time for online diagnosis. This intelligent tongue colour diagnosis system can be employed as an assisted tool for the practitioners and medical doctors to diagnose any unknown tongue colour image during their practice.

## Figures and Tables

**Algorithm 1:**
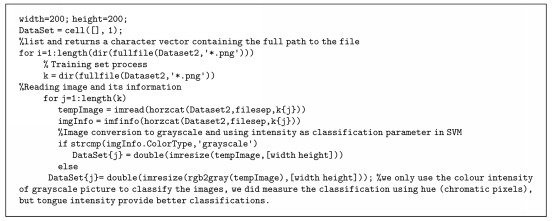
Algorithm 1: Pseudocodes of training input setting in SVM.

**Algorithm 2:**
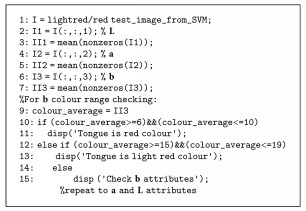
Algorithm 2: Pseudocodes of red colour ranges.

**Table 1:** Red colour range for red and light red tongues.

Tongue colour	*Lab* colour range		
	*L* ^∗^	*a* ^∗^	*b* ^∗^
Red	*L* ^∗^ < 56	32 ≤ *a*^∗^ ≤ 39	6 ≤ *b*^∗^ ≤ 10
Light red	*L* ^∗^ ≥ 56	23 ≤ *a*^∗^ ≤ 27	15 ≤ *b*^∗^ ≤ 19

**Table 2:** Comparison of average classification accuracy and execution time of several algorithms using same database specifications.

Method	Technique/kernel	Accuracy (%)	Execution time (s)
Conventional SVM (only SVM) [11, 13, 27, 28, 42]	RBF	50	219
	Polynomial	50	187
	Linear	57	249
	Quadratic	74	187

Proposed SVM with clustering identifiers (SVM + *k*-means)	RBF	63	166
	Polynomial	50	172
	Linear	89	149
	Quadratic	50	151

Neural network [18, 24]	Conventional	70.6	652

**Table 3:** Comparison of red colour range's performance in classification.

*Lab* colour space attributes	Accuracy
Only chromatic attribute range (*a*^∗^, *b*^∗^)	63%
Both chromatic and luminance attribute range (*L*^∗^, *a*^∗^, *b*^∗^)	95%
